# Renal Angiomyolipoma Associated with Inferior Vena Cava Thrombus

**DOI:** 10.1155/2009/789078

**Published:** 2009-12-16

**Authors:** Xavier Durand, Raphaelle Renard-Penna, Eva Comperat, Marc-Olivier Bitker, Francois Richard

**Affiliations:** ^1^Department of Urology, Pitié Salpetriere University Hospital, 75013, Paris, France; ^2^Department of Radiology, Pitié Salpetriere University Hospital, 75013, Paris, France; ^3^Department of Pathology, Pitié Salpetriere University Hospital, 75013, Paris, France

## Abstract

A 57-year-old woman was found to have an inferior vena cava involvement of a known sinusal angiomyolipoma incompletely resected three years beforehand. Intravascular extension into the IVC of angiomyolipoma has rarely been reported. We present a new case and reconsider the literature about this uncommon complication of a benign renal tumor.

## 1. Introduction

Renal angiomyolipoma (AML) is typically a solid lesion, composed of adipose tissue, dystrophic vessels, and smooth muscle cells, lacking an epithelial component [1]. This unusual benign tumor accounts for 3% of all solid kidney tumors, with a female predominance (sex ratio 4 : 11) [2]. It can occur sporadically or in association with phacomatoses (20%) [3].Even if sarcomatous degeneration has been described and excepted the epithelioid form, AML has a benign course and a slow local development.AML may remain asymptomatic or become complicated typically from tumour growth or haemorrhage beyond a 40 mm size. However, AML can exceptionally involve the renal vein and the inferior vena cava as a tumour thrombus. Such cases have been described fifteen times in literature [2, 4–8]. We report back a new case.

## 2. Case Presentation

A 57-years-old patient is submitted to scanographic survey after multiple and bilateral renal tumorectomies of asymptomatic lesions. Three years beforehand, a tumorectomy had been performed at the inferior pole of the left kidney. 

**Figure 1:**
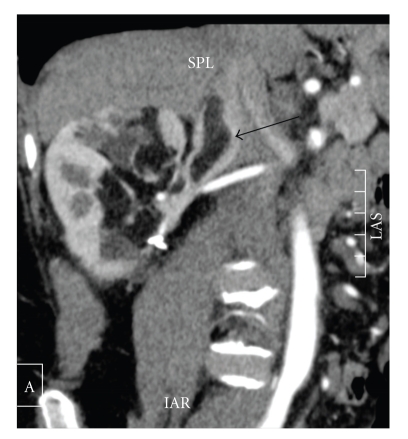
Computed tomography scan demonstrating right, fat containing, renal mass with renal vein and vena cava involvement (arrow).

One year later, four tumorectomies had been performed on the right kidney, one of which is incomplete in the renal sinus, due to the relationships between the lesion and the vascular pedicle. Pathological analysis of all specimens showed renal angimyolipomas without any sign of malignancy. Tumoral cells were weakly marqued with PS 100 and HMB 25. Two years later, an abdominal computerized tomography control revealed a 45 mm fatty mass of the right renal sinus extended to the renal vein and the IVC as a tumour thrombus (Figure 1). 

Magnetic resonance imaging precised the thrombus features and extended 30 mm under the hepatic veins.

**Figure 2:**
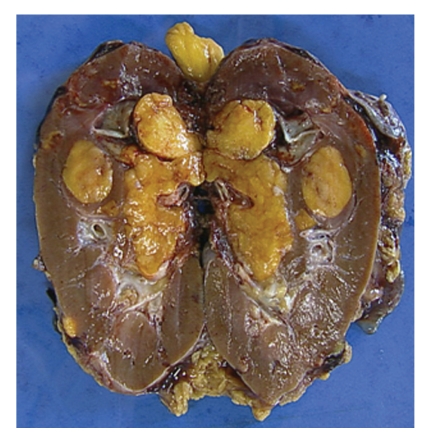
Gross specimen of right kidney with fatty sinusal tumor and yellowish tumor thrombus.

Right nephrectomy and thrombectomy with a short cavotomy is performed. Pathological analysis confirmed the diagnosis of conventional angiomyolipoma with a 60 mm caval fatty thrombus (Figure 2). The patient was discharge home on postoperative day 10.

## 3. Discussion

Angiomyoplipoma is a rare benign tumor of the group of perivascular epithelioid cell tumors [9]. The conventional AML usually follows benign course of slow growth and local development. The epithelioid form of angiomyolipoma is a singular entity, with poor prognosis, which contains no adipose tissue and can be mistaken for renal cell carcinoma. Conventional AML is often diagnosed on symptomatic complications such as tumor growth or haemorrhage. The diagnosis is made by CT, showing adipose tissue areas with negative density (−20 to −100 HU) in the lesion. However, 5% of angiomyolipomas do not have any fatty tissue visible on CT scan. T1-weighted sequence MRI shows a high-intensity signal which becomes hypointense on saturation-fat sequence [10]. Fifteen cases of renal vein or inferior vena cava involvements have been reported between 1985 [4] and 2006 [8]. The tumors were sometimes bilateral, overtaking readily 100 mm, reaching sometimes the right atrial cavity [5, 8] and were associated with phacomatoses in two cases [6]. Most of AML cases require simple monitoring and have to be surgically removed when haemorrhage occurs. Nephron sparing surgery is the best option regard for these tumors which may evolve bilateraly. Transarterial selective embolization (TAE) is the therapy of first choice for retroperitoneal bleeding in case of AML rupture, to avoid nephrectomy. TAE is also recommended in case of big sized AML, larger than 40 mm, to prevent complication, especially in a unilateral residual kidney. Embolization of the tumor vasculature (aneurysms, tortuous dysmorphic arteries) results in reduction of the tumor diameter and conservation of normal parenchyma [11]. It would have been a good option for the right kidney of our patient, better than incomplete resection in a nephron sparing surgical procedure. The initial conservative approach is important because of the underlying possibility of vein involvement which may necessitate total nephrectomy. Agressive forms of AML do exist: bilateral, with fast growth, multiple localizations, and vein involvement. It has to be known even apart from tuberous sclerosis.

## 4. Conclusion

This observation highlights the problem of angiomyolipomas' survey. An annual abdominal CT is advisable for the initial 2-3 years on order to assess the growth velocity of the lesion before deciding on the subsequent rate of followup checks.

## References

[1] Nelson CP, Sanda MG (2002). Contemporary diagnosis and management of renal angiomyolipoma. *Journal of Urology*.

[2] Gamé X, Soulié M, Moussouni S (2003). Renal angiomyolipoma associated with rapid enlargment and inferior vena caval tumor thrombus. *Journal of Urology*.

[3] Pfister C, Thoumas D, Fauquet I (2002). Diagnostic and therapeutic approach of angiomyolipoma. *Progres en Urologie*.

[4] Brantley RE, Mashni JW, Bethards RE, Chernys AE, Chung WM (1985). Computerized tomographic demonstration of inferior vena caval tumor thrombus from renal angiomyolipoma. *Journal of Urology*.

[5] Ito H, Nakashima S, Toma H, Misaki T (1999). Renal angiomyolipoma associated with inferior vena caval and right atrial thrombus. *Journal of Urology*.

[6] Toda F, Okuda H, Kondo N (1999). Renal angiomyolipoma with a tumor thrombus extending into the right atrium—a case report. *Nippon Hinyokika Gakkai Zasshi*.

[7] Wilson SS, Clark PE, Stein JP (2002). Angiomyolipoma with vena caval extension. *Urology*.

[8] Haritharan T, Sritharan S, Bhimji S (2006). Renal angiomyolipoma with inferior vena caval involvement. *Medical Journal of Malaysia*.

[9] Compérat E, Camparo P, Vieillefond A (2006). WHO classification 2004: tumors of the kidneys. *Journal de Radiologie*.

[10] Israel GM, Hindman N, Hecht E, Krinsky G (2005). The use of opposed-phase chemical shift MRI in the diagnosis of renal angiomyolipomas. *American Journal of Roentgenology*.

[11] Kothary N, Soulen MC, Clark TWI (2005). Renal angiomyolipoma: long-term results after arterial embolization. *Journal of Vascular and Interventional Radiology*.

